# macpie: Scalable workflow for high-throughput transcriptomic profiling

**DOI:** 10.1016/j.csbj.2025.11.002

**Published:** 2025-11-07

**Authors:** Nenad Bartonicek, Xin Liu, Laura Twomey, Michelle Meier, Richard Lupat, Stuart Craig, David Yoannidis, Jason Li, Tim Semple, Kaylene J. Simpson, Mark X. Li, Susanne Ramm

**Affiliations:** a Peter MacCallum Cancer Centre, Melbourne, VIC 3000, Australia; b Sir Peter MacCallum Department of Oncology, The University of Melbourne, VIC 3010, Australia; c Victorian Centre for Functional Genomics, Peter MacCallum Cancer Centre, Melbourne, VIC 3000, Australia; d Department of Biochemistry and Pharmacology, The University of Melbourne, Victoria 3010, Australia; e Bioinformatics Core Facility, Peter MacCallum Cancer Centre, Melbourne, VIC 3000, Australia

**Keywords:** High-throughput transcriptomics, Plate-based screens, Data integration, Software

## Abstract

High-throughput transcriptomic profiling (HTTr) enables scalable characterisation of transcriptional responses to chemical and genetic perturbations. While plate-based technologies such as MAC-Seq, TempO-seq and PLATE-seq have made HTTr more accessible, they pose unique computational challenges for data modelling and integration across modalities. We present *macpie*, an R package designed to streamline the analysis of HTTr data from plate-based screens. Built on the tidySeurat framework, *macpie* streamlines the entire analytical pipeline from preprocessing and quality control to pathway enrichment, chemical feature extraction, and multimodal data integration. The package incorporates multiple statistical frameworks and uses parallelisation for scalability. By leveraging Docker and Nextflow, macpie ensures reproducibility and ease of use for transcriptome-wide screening.

**Data availability:**

All code and example datasets used in this study are available in the *macpie* GitHub repository (https://github.com/PMCC-BioinformaticsCore/macpie). Additional data supporting the findings of this study are available from the corresponding author upon reasonable request.

## Graphical Abstract

**Figure:**
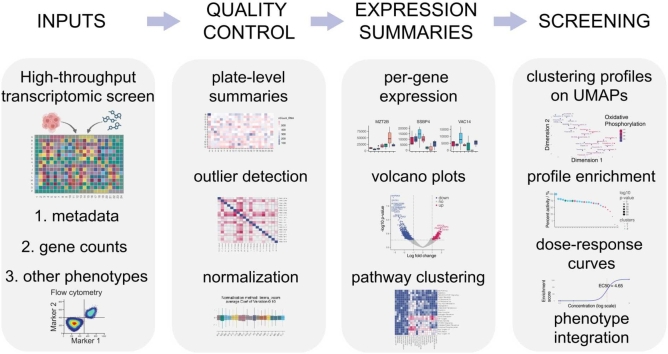

## 1 Introduction

High-throughput screening (HTS) platforms are widely used in modern biomedical research, supporting a broad range of applications from functional genomics (e.g., CRISPR or RNAi screens), biomarker identification, drug discovery, to toxicology [1], [2], [3], [4]. Early HTS approaches commonly reduced complex cellular phenotypes to a limited set of features such as proliferation rates, morphological changes or biomarker abundance, and were gradually replaced with multi-feature, high-content screening (HCS) [5]. More recently, high-throughput transcriptomic (HTTr) profiling has emerged as an increasingly accessible and scalable extension of HCS, capturing dynamic cellular states in response to chemical or genetic perturbations [6], [7], [8].

Several technologies support HTTr, including DRUG-seq [9], TempO-seq [10], L1000 [6], PLATE-seq [11], and commercial platforms such as InSphero’s Organoid DRUG-seq [12]. MAC-Seq (Multiplexed Analysis of Cells) was introduced as a cost-effective HTTr method that eliminates RNA extraction steps and supports integration with suspension cells, flow cytometry readouts and high-content imaging for adherent, 3D matrix embedded cell models, as well as complex co-culture systems [13]. Even though these platforms overcome considerable technical barriers related to plate-based workflows, they also present unique analytical challenges.

High-throughput transcriptomic platforms generate datasets that fall outside the scope of existing computational workflows. Compared to conventional RNA-seq, HTTr datasets often involve complex experimental designs with a larger number of perturbations and higher potential for latent batch effects. The limited input material and small cell numbers frequently result in zero-inflated count distributions, resembling those seen in single-cell RNA-seq (scRNA-seq) [14]. However, HTTr datasets generally lack sufficient samples to support statistical models developed for scRNA-seq, presenting unique analytical challenges.

Current HTTr analytical frameworks have mostly been built to address toxicology analyses and are available as standalone applications (*BMDExpress-2* [15]) or partial workflows in R for quality control (e.g. *httrpl* [8]), analysis and visualisation (e.g. DRUG-seq [16]) or modelling of compound concentration-response curves (e.g. *tcplfit2* [17], and *bmd* [18]). A complete, modular, and scalable R-based framework integrating both bioinformatics and cheminformatics components remains absent. To address this gap, we introduce *macpie*, an R package for the comprehensive analysis of HTTr data from plate-based sequencing technologies.

## 2 Implementation

The *macpie* package was developed for analysis and visualisation of HTTr data, particularly for large, 384-well plate-based screens. The workflow is based on the *tidySeurat* framework that combines properties of *Seurat* and *tidyverse* objects, benefitting from their respective functionalities. *Seurat* is a widely used R package for complete analysis of transcriptomic data from single cell experiments [19], including QC, dimensionality reduction, clustering, marker selection and data integration. The *tidyverse* collection of R packages is the standard in R-based data analytics [20] for preprocessing, modelling and visualisation of complex data structures. *macpie* is compatible with R version > 4.3.3 and is available as a Docker container to ensure reproducibility and ease of installation. Key steps of the analytical workflow are outlined in the package vignettes.

### 2.1 Data preprocessing

*macpie* requires gene expression matrices and corresponding sample metadata sheet as inputs*.* Matrices can be imported from the standard sparse matrix format, as generated by Cell Ranger [22] or STARsolo [23] from raw FASTQ files. The sample metadata sheet must contain a minimal set of standard columns as outlined in the documentation, ensuring accurate mapping of expression values to sample annotation via sample barcodes, and can be further populated by descriptors of samples or perturbations. To simplify and standardise data preprocessing from FASTQ files, we provide a companion Nextflow pipeline, available at https://github.com/PMCC-BioinformaticsCore/dinoflow. *macpie* currently supports data from *Homo sapiens* and *Mus musculus*.

### 2.2 Quality control

**Fig. 1:**
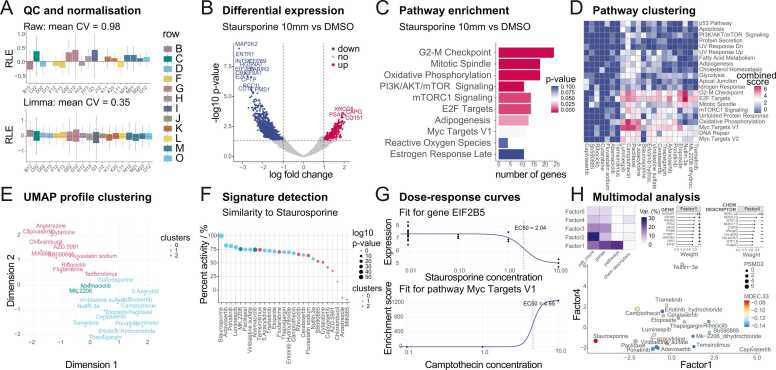
**Overview of macpie key functionalities.** A. Relative log expression (RLE) plots to estimate sources of variability during quality control and normalisation. B. Volcano plot showing differential gene expression (DE) following Staurosporine 10 µM treatment vs DMSO vehicle control. C. Pathway enrichment heatmap derived from DE analysis. D. Aggregated pathway visualisation, combining pathway enrichment results across replicates. E. UMAP dimensionality reduction based on DE of treatments compared to DMSO. F. Point plot for signature detection, based on the enrichment of top 500 genes from an existing perturbation profile, as measured by Normalised Enrichment Score (NES). G. Dose-response curves at the gene level (top) and pathway level (bottom), with EC50 values indicated by dashed red lines. H. Multimodal analysis using MOFA [21]. Upper panels describe latent factors of a model that combines cell viability, gene expression, pathway enrichment and chemical properties of compounds. Lower panel shows low-dimensional representation of samples coloured by molecular descriptor MDEC.33, with data point size defined by expression of by PSMD2 gene, a cancer growth promoter.

Given the large-scale nature of HTTr transcription-based screens, quality control is essential to ensure data integrity and minimise technical artefacts (Fig. 1A). The QC workflow begins with metadata validation, as screens involve complex, often manually entered combinations of treatments. To streamline this step, we simplified inspection of large numbers of experimental variables for potential errors or inconsistencies, including automated checks for missing values and special characters. Next, *macpie* provides a set of QC tools that assess various properties of read distribution across genes and experimental conditions: read depth, variability, variance decomposition, outlier detection and normalisation. Relative log expression (RLE) plots are especially effective at visualising impacts of filtering cutoffs and various normalisations methods, while quantifying differences using the average coefficient of covariation (Fig. 1A).

### 2.3 Transcriptomic workflow

Following QC, *macpie* supports two principal analytical modes: single-treatment and multi-treatment analysis. At the single-treatment level, *macpie* facilitates characterisation of transcriptional responses to individual perturbations. Differential expression analysis is implemented with support for multiple statistical frameworks, including Seurat [19], DESeq2 [24], edgeR [25], RUVSeq [26] and ZINB-WaVE [27], allowing users to choose between models based on their dataset characteristics. Differentially expressed genes (DEGs) can be visualised on volcano plots (Fig. 1B), placed in biological context through gene set enrichment and pathway analysis tools (Fig. 1C), and samples visualised on multidimensional scaling (MDS) and Uniform Manifold Approximation and Projection (UMAP) plots. In addition, *macpie* offers a multi-treatment analysis framework, enabling users to compare transcriptional profiles across numerous perturbations in parallel. This mode automates the computation of DEGs and the enrichment analysis via standardised pipelines, producing summaries of results at gene and pathway levels (Fig. 1D-F). All multi-treatment analyses are parallelised to optimise runtime and computational efficiency.

### 2.4 Screen-level analyses

To explore higher-order effects across perturbations and integrate them with user-provided external annotations such as cell counts of surface markers, the *macpie* workflow streamlines several visualisations and analyses. First, *macpie* simplifies UMAP dimensionality reduction, enabling users to cluster experimental factors by the similarity of their transcriptional responses (Fig. 1E). In addition, *macpie* supports cheminformatics workflows by extracting SMILES strings from compound names, computing and filtering molecular descriptors (e.g. physicochemical properties and fingerprints) or calculating half-maximal effective concentrations for genes or pathways (Fig. 1G). Automated compound recognition is performed via the function *compute_smiles*(), which queries PubChem through the R package *webchem* to retrieve isomeric SMILES. Users may alternatively provide curated identifiers or verified SMILES directly in the metadata prior to running *compute*_*chem*_*descriptors*(), ensuring flexibility and reproducibility across datasets. Finally, *macpie* allows data integration with Multi-Omics Factor Analysis (MOFA) [21], facilitating unsupervised decomposition of variance across different data modalities, revealing latent factors driving biological or technical variation (Fig. 1H).

### 2.5 Benchmarking of statistical models and other HTTr methods

**Fig. 2:**
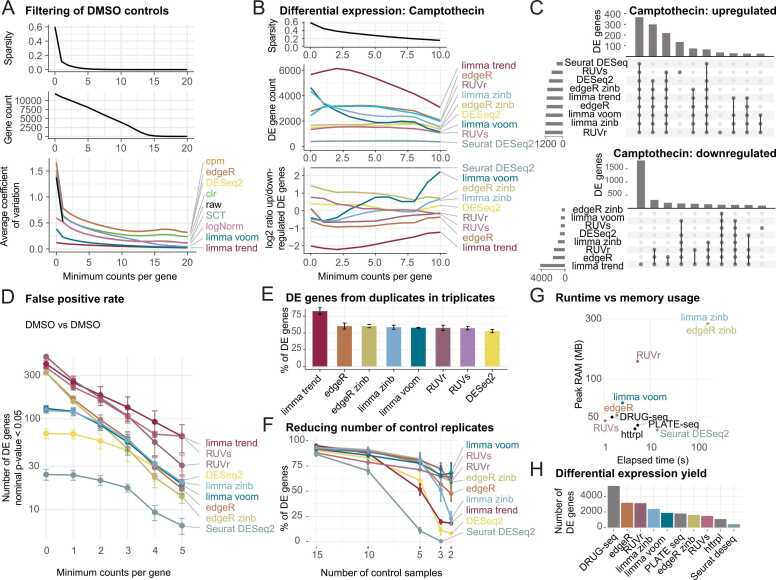
**Performance of gene filtering, statistical methods and HTTr methods.** A. Effects of filtering reads by minimum read count in DMSO controls. Panels show changes in sparsity (top), number of detected genes (middle), and average coefficient of variation from RLE plots of normalised counts across methods (bottom). B. Impact of read-count filtering on differential-expression models in Camptothecin vs DMSO. Top: sparsity levels. Middle: number of differentially expressed (DE) genes with |log₂ fold change| > 1 and FDR < 0.05. Bottom: ratio of up- to down-regulated genes. Values for Seurat DESeq represent positive infinity, due to a lack of downregulated genes. C. UpSet plots showing overlap of DE genes across statistical models for Camptothecin vs DMSO (top = up-regulated; bottom = down-regulated) at a filtering cutoff of five reads per gene per group. D. Estimated false-positive rates based on comparison of two random DMSO groups (n = 3 per group; total n = 8 per group). Lines show the number of genes with nominal p < 0.05 and |log₂ fold change| > 1. Error bars represent standard error. E. Bar graph with percent of DE genes from duplicates (Camptothecin vs DMSO, 3x n = 2) overlapping DE genes from a triplicate (Camptothecin vs DMSO, 1x n = 3) across statistical models, |log2FC= >1 and FDR< 0.05. Error bars represent standard error (n = 3). F. Relationship between the number of control replicates and DE gene yield across statistical models. Error bars represent standard error (n = 3). G. Run time and memory usage for DRUG-seq (limma trend), PLATE-seq and httrpl, compared to other statistical models available through macpie. H. Number of DE genes found through statistical methods available through macpie or external workflows for Camptothecin vs DMSO, |log2FC= >1 and FDR< 0.05.

To assess the performance and robustness of statistical frameworks implemented in *macpie*, we benchmarked multiple normalisation and differential-expression (DE) pipelines (Fig. 2). We standardly use relative log expression (RLE) plots of normalised counts to estimate batch effects as well as quality of controls (Fig. 1A). Filtering of low-count genes reduced the sparsity of expression matrices, increasing the minimum read threshold eliminated low-abundance transcripts, and leading to more stable distributions in RLE plots. This resulted in lower average coefficients of variation across normalisation methods, and other metrices across datasets (Fig. 2A, Fig. S1-2). *DESeq* and *edgeR*, which report normalised counts based on adjusted counts per million (CPM), performed identically to CPM alone.

We next examined how filtering affected DE analyses across models: *DESeq2*, *edgeR*, *limma voom*, *limma trend*, *RUVSeq*, and *ZINB-WaVE* in combination with *edgeR* (*edgeR zinb*) and *limma voom* (*limma zinb*). In *Camptothecin (n = 3) vs DMSO (n = 19)* comparisons, filtering generally decreased the number of detected DE genes, but improved concordance between methods (Fig. 2B, Fig. S3). The ratio of up- to down-regulated genes remained consistent across filtering thresholds, indicating that filtering primarily affects sensitivity rather than directionality of expression change. Overlap analysis using UpSet plots showed that DE calls from *limma voom*, *edgeR*, and *DESeq2* shared a large common core of up- and down-regulated genes, with a smaller impact of ZINB-WaVE weights at this filtering threshold. Distribution of downregulated DE genes across methods was less consistent, indicating difficulties with estimation of lowly expressed genes for some of the datasets (Fig. 2C, Fig. S4).

To estimate false-positive rates, we compared two random groups of DMSO replicates (2 x *n = 8*). No methods produced significant genes with |log₂ fold-change| > 1 and FDR< 0.05, but the distribution of nominally significant genes at *p* < 0.05 confirmed that filtering reduces Type I error rates (Fig. 2D, Fig. S5). Decreasing the number of sample replicates from three to two had a profound effect on the number of DE genes, which decreased about 20–40 % across methods (Fig. 2E, Fig. S6). Similarly, removing replicates decreased DE yield across all models, with more prominent diminishing returns when replicate count was below five (Fig. 2F, Fig. S6).

Finally, we compared performance of *macpie* statistical models with three other HTTr methods: *PLATE-seq*, *httrpl* and *DRUG-seq*, starting from matrices of gene counts (Fig. 2G-H). *PLATE-seq* utilises *DESeq2*, httrpl uses *DESeq2* with shrinkage, and *DRUG-seq* uses *limma trend* as their statistical models*.* Out of models available through *macpie*, *edgeR* and *limma voom* were comparable in performance to *DRUG-seq*’s *limma trend*, and performed faster and with lower memory footprint than *PLATE-seq* and *httrpl*. Comparison of total DE yield across methods and datasets (Fig. 2H) demonstrated that most *macpie* methods provide a larger number of DE genes compared to *httrpl*, and similar number to *PLATE-seq*. *DRUG-seq*’s *limma trend* provided the largest number of DE genes, but as previously observed, these results were not concordant with other methods, especially in downregulated genes (Fig. 2C).

## 3 Conclusion

*macpie* offers a robust, well-structured and flexible R-based framework for the analysis of high-throughput transcriptomic screens. By standardizing preprocessing and integrating diverse statistical methods, it enables consistent and interpretable analysis of complex datasets. A complementary Nextflow pipeline for raw data preprocessing and containerised environment support were developed to provide scalability and reproducibility required for large-scale screening efforts. The modular architecture and emphasis on portability were designed to encourage future community contributions to *macpie*. We successfully applied *macpie* to other external HTTr datasets (vignette “cross_platform_compatibility”), confirming its robustness across different platforms, cell types, and perturbations.

Based on our benchmarking analysis, we recommend assessing datasets for zero inflation and evaluating how gene-filtering thresholds influence the chosen statistical model (see vignette “Assessing zero-inflation”) The choice of normalisation and differential expression method can substantially affect both the number and direction of DE genes. This variability is particularly pronounced for down-regulated genes following treatment with potent cell-killing compounds, where different models yield divergent results. To ensure robust inference, we advise including a sufficient number of DMSO controls and systematically testing how sample variability and control selection impact statistical outcomes. *macpie* simplifies such exploratory analyses, enabling users to efficiently trial and compare alternative models within a unified workflow.

Beyond its technical framework, *macpie* benefits researchers by providing an end-to-end, reproducible workflow that bridges transcriptomic data processing, quality control, and downstream analysis within a single environment. This integration allows experimental and computational scientists to rapidly prioritise perturbations based on transcriptional or phenotypic responses and to explore drug–gene relationships through built-in cheminformatics modules. Clinicians and translational researchers can further leverage *macpie* to identify mechanistic signatures and treatment-specific transcriptional responses, facilitating the discovery of biomarkers and candidate compounds for validation.

As multimodal profiling becomes standard for characterising cellular responses, *macpie* provides a foundation for integrating transcriptomic and phenotypic data with chemical or biological properties of treatments. Future developments will incorporate additional omics layers, such as surface proteomics, secretomes, long-read sequencing and cellular barcoding [28], facilitating more comprehensive systems-level analyses.

## 4 Methods

Differential expression (DE) analyses were performed using the *compute_single_de*(). Limma-based methods used *TMMwsp* normalisation for data with a high proportion of zeros. *edgeR* was used with negative binomial generalised linear models with quasi-likelihood F-tests (*glmQLFit*). Seurat DE was performed with Seurat’s rank-sum test (Wilcoxon). Normalised *RUVSeq* counts were reanalysed with *edgeR* GLMs. For sparse or zero-inflated datasets, *ZINB*-*WaVE* was used to estimate observation-level weights under a zero-inflated negative binomial (*ZINB*) model (K = 2, ε = 12,000). These weights were incorporated into *edgeR* (*edgeR*-*ZINB*) or limma-voom (*limma*-*ZINB*) pipelines.

Benchmarking was performed on *macpie* gene count matrix available with the package, filtered for genes with at least 5 reads in at least 2 sample groups. Parameters of *limma trend* were adjusted to match DRUG-seq protocol. Package *httrpl* was benchmarked using function *runDESeq2*.

## Author contributions

N.B., X.L., M.X.L and S.R. conceived, designed and implemented the study and co-wrote the manuscript. N.B. and X.L. developed the *macpie* R package and performed analyses. N.B., X.L. and R.L. implemented the Nextflow pipeline L.T. and M.M. contributed to software development, visualisation and design. R.L. provided computational and software support. S.C., and D.Y. provided technical expertise for generation of experimental data. J.L., T.S., and K.J.S. supervised the project. All authors read and approved the final manuscript.

## Funding

This work was supported by a Peter Mac Foundation Grant awarded to S.R.

## CRediT authorship contribution statement

**Mark X Li:** Writing – review & editing, Writing – original draft, Visualization, Supervision, Software, Resources, Project administration, Formal analysis, Conceptualization. **Nenad Bartonicek:** Writing – review & editing, Writing – original draft, Visualization, Validation, Software, Methodology, Formal analysis, Conceptualization. **Tim Semple:** Writing – review & editing, Supervision. **Kaylene J Simpson:** Writing – review & editing, Supervision, Resources, Project administration. **David Yoannidis:** Resources, Conceptualization. **Jason Li:** Writing – review & editing, Supervision. **Richard Lupat:** Software, Resources, Methodology. **Stuart Craig:** Resources, Conceptualization. **Laura Twomey:** Visualization, Software, Methodology, Conceptualization. **Michelle Meier:** Visualization, Software, Formal analysis, Conceptualization. **Susanne Ramm:** Writing – review & editing, Visualization, Supervision, Software, Project administration, Funding acquisition, Conceptualization. **Xin Liu:** Writing – original draft, Visualization, Validation, Software, Resources, Methodology, Formal analysis, Data curation, Conceptualization.

## Declaration of Competing Interest

The authors declare no conflict of interest.

## Data Availability

The R package *macpie* is freely available at https://github.com/PMCC-BioinformaticsCore/macpie, with images of the working environment hosted at Docker Hub: xliu81/macpie. A companion Nextflow pipeline for preprocessing from FASTQ files is available at https://github.com/PMCC-BioinformaticsCore/dinoflow.
