# Supplementary material for: macpie: Scalable workflow for high-throughput transcriptomic profiling
Source: Comput Struct Biotechnol J. 2025 Nov 7;27:5129–34. doi: 10.1016/j.csbj.2025.11.002 (PMC12664655; doi:10.1016/j.csbj.2025.11.002)
Supplement: Supplementary file 1 — Supplementary material [file mmc1.pdf]

Figure S1.

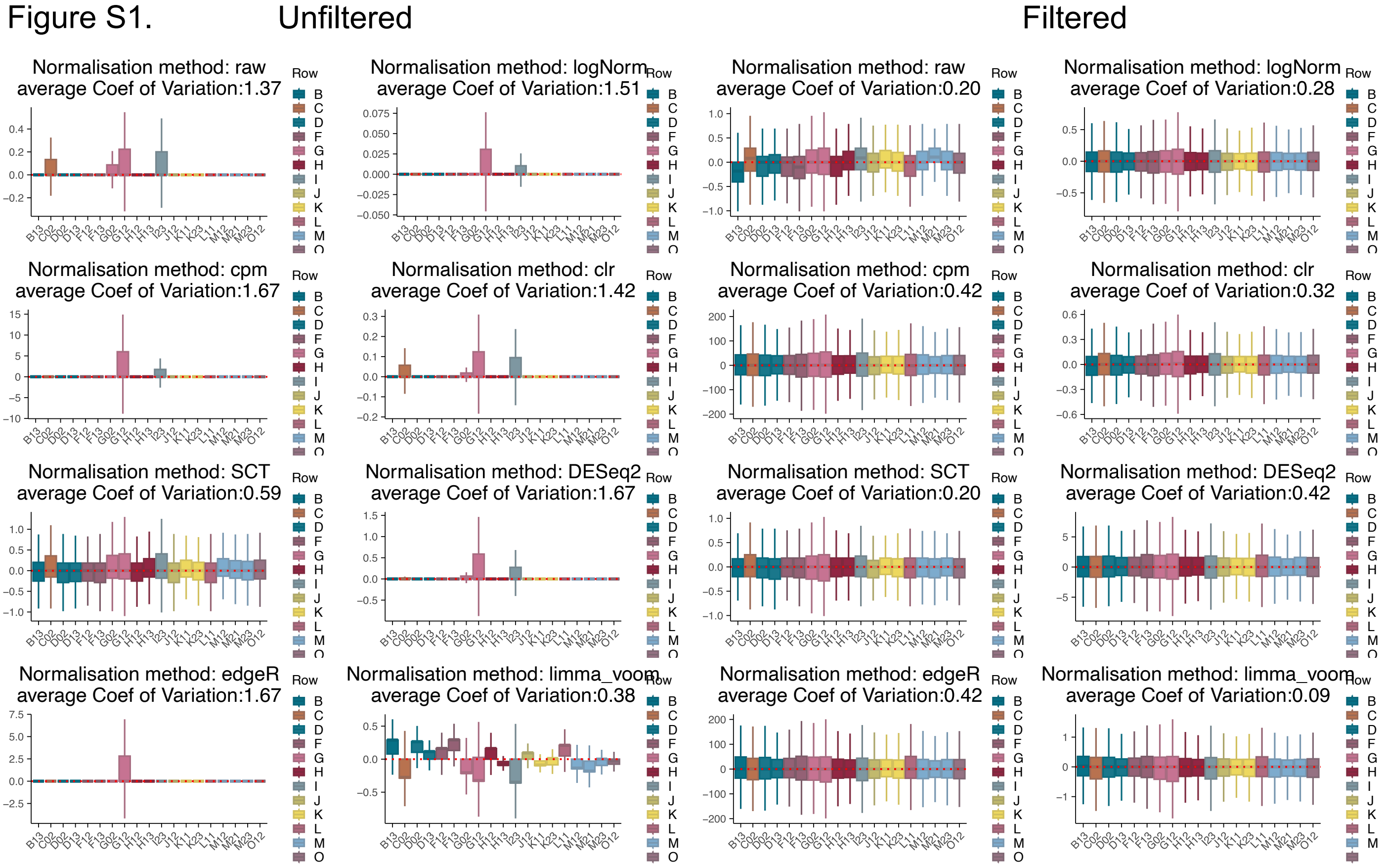

Supplementary Figure S1. Comparison of normalisation methods and their effect on variability across DMSO samples for unfiltered and filtered (n=10) reads. Relative log enrichment (RLE) plots show the distribution of mean-centred expression values for individual samples, colored by row. Panels represent: raw counts, log-normalisation, counts per million (CPM), centred log-ratio (CLR), SCTransform (SCT), DESeq2, edgeR, and limma-voom from unfiltered or filtered data (bottom row). The average coefficient of variation (CV) for each method is indicated within the panel.

Figure S2.

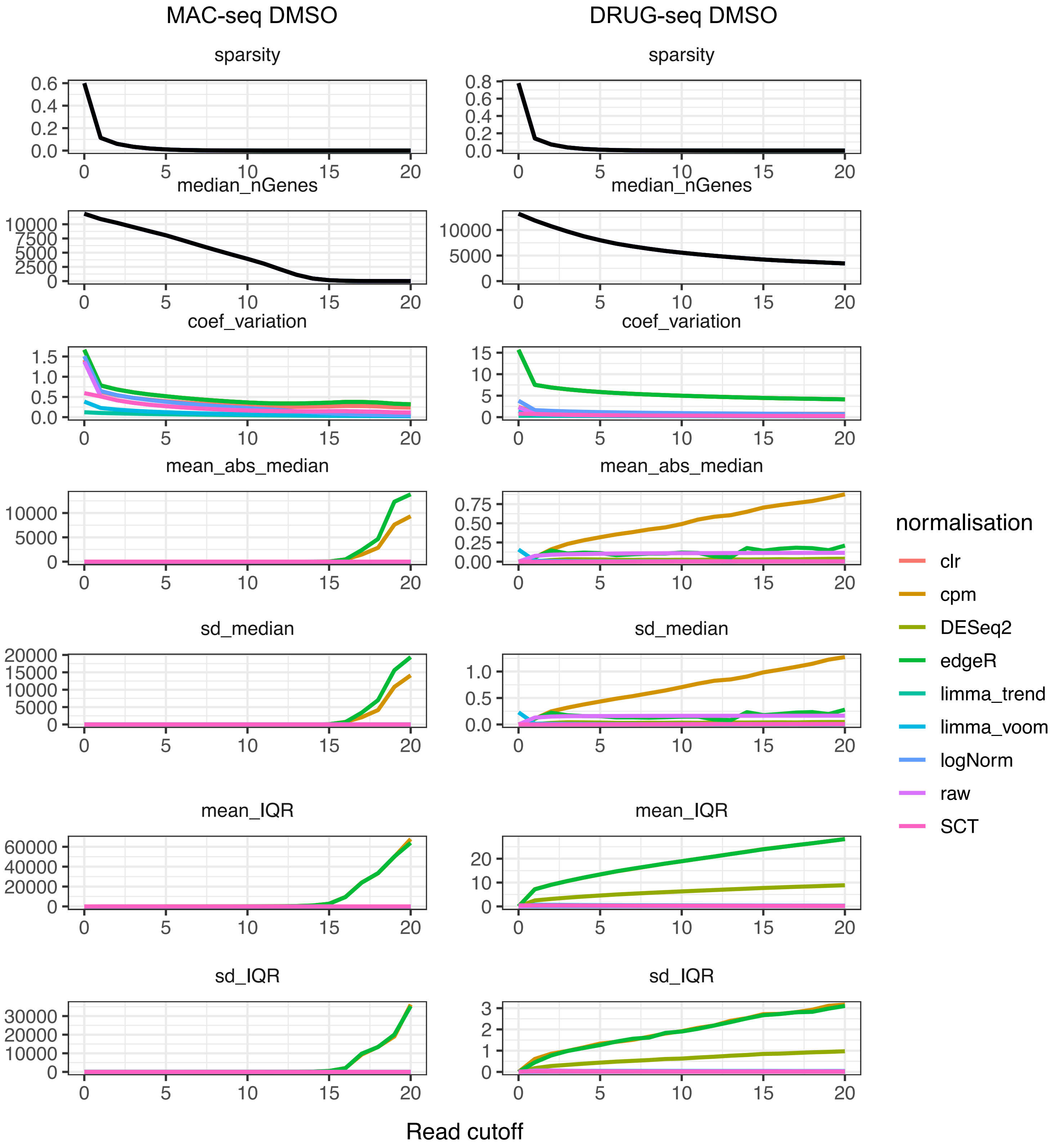

Supplementary Figure S2. Effect of read cutoff and normalisation methods on RLE plots. Each panel summarises quality control metrics across increasing read cutoffs for DMSO from MAC-seq (left) or DRUG-seq (right) datasets, comparing eight normalization methods (raw, logNorm, CPM, CLR, SCTransform, DESeq2, edgeR, limma-trend, and limma-voom). Metrics include: sparsity (fraction of zero counts), median number of detected genes per sample, coefficient of variation (CV), mean absolute deviation from the median, standard deviation of the median, mean interquartile range (IQR), and standard deviation of the IQR.

Figure S3.

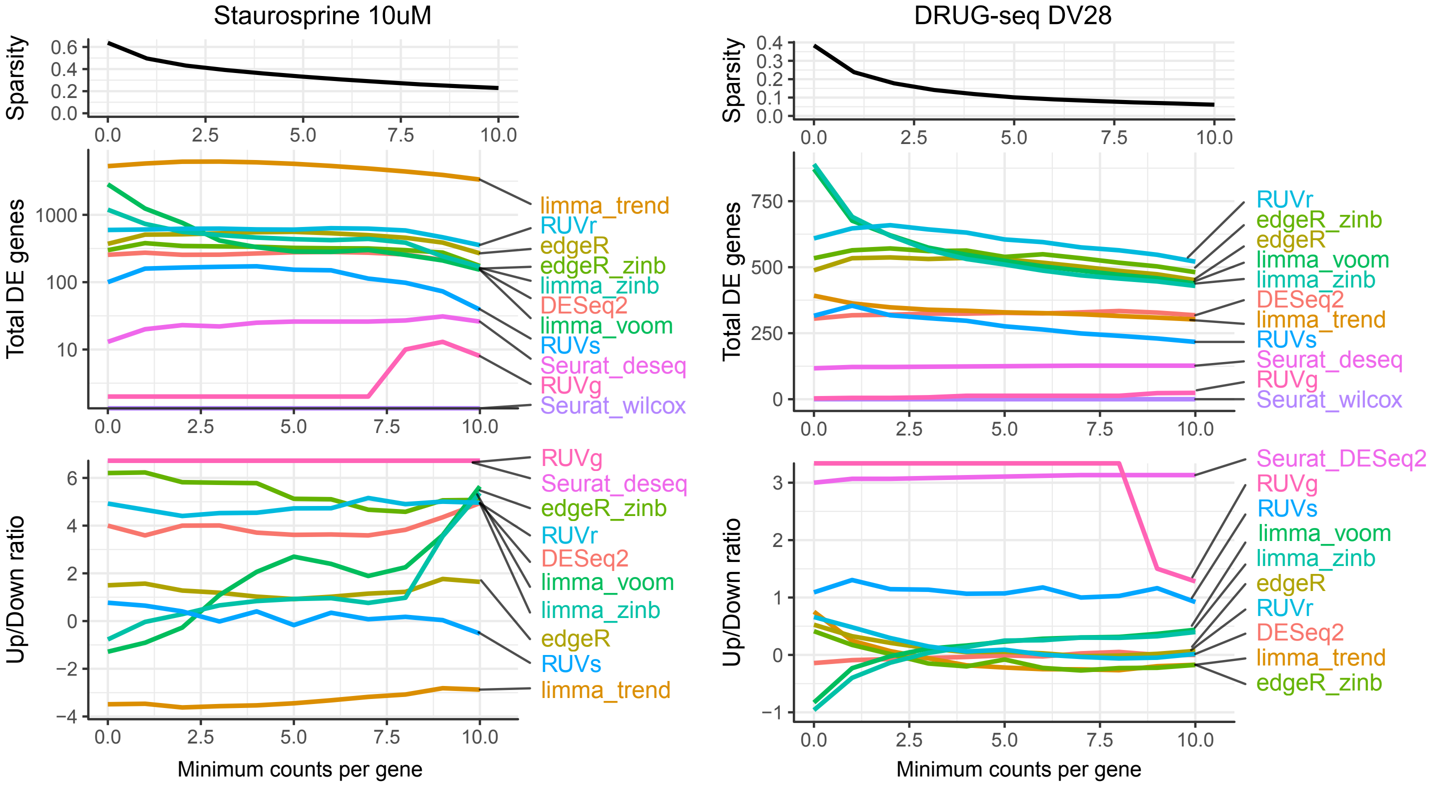

Supplementary Figure S3. Comparison of differential expression outcomes across modeling methods under varying read cutoffs. The plots show the effect of increasing the minimum read count per gene on key metrics of differential expression for the Staurosporine 10 μM and DRUG-seq DV28 dataset. Metrics include sparsity (fraction of zero-count genes), total number of differentially expressed (DE) genes, and log2 ratio of up vs down-regulated genes.

Figure S4.

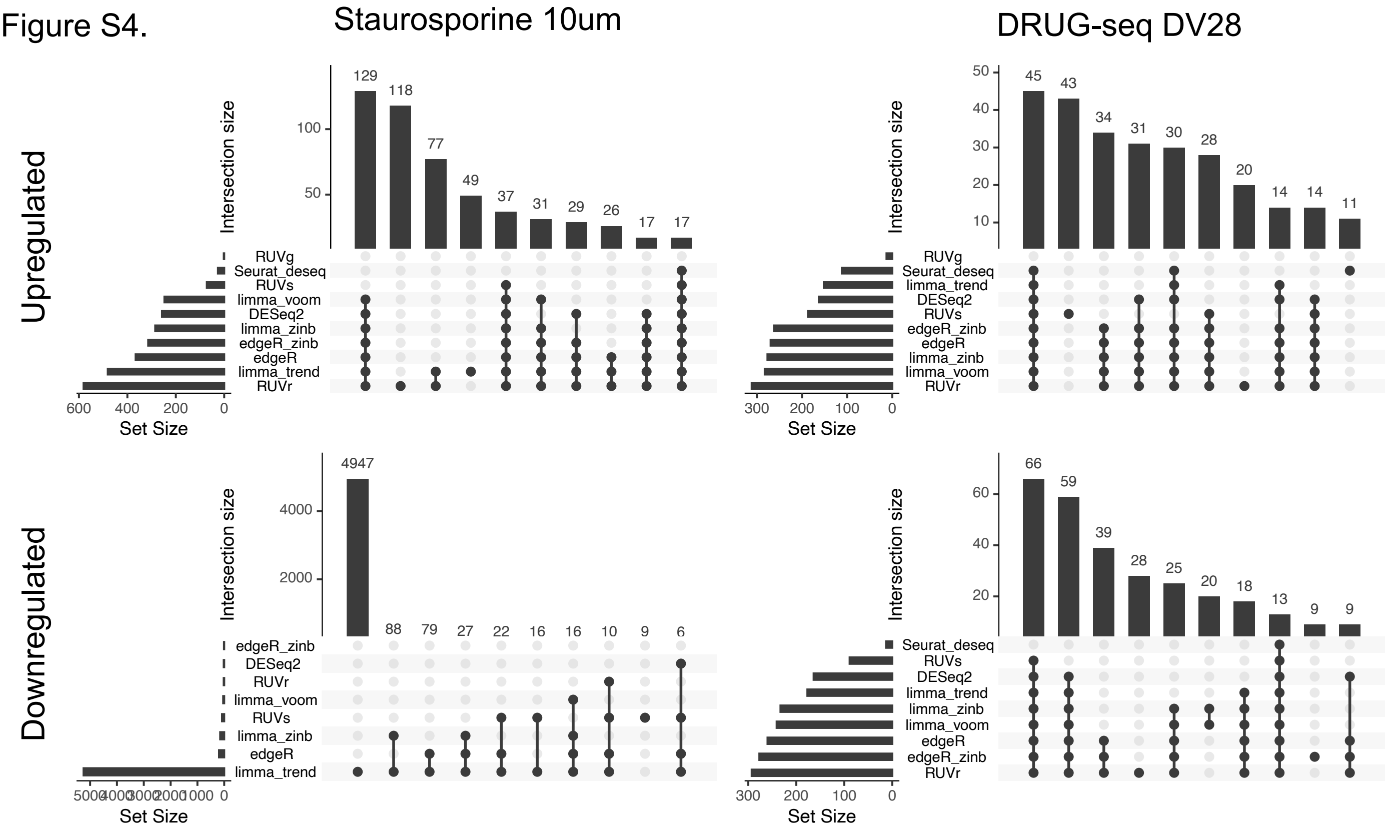

Supplementary Figure S4. Overlap of significantly differentially expressed genes across normalization and modeling methods. UpSet plots show the intersections of upregulated (top) and downregulated (bottom) genes identified by multiple statistical models in the Staurosporine 10 μM and DRUG-seq DV28 dataset. Methods compared include DESeq2, edgeR, edgeR_zinb, limma-trend, limma-voom, limma_zinb, RUVg, RUVr, RUVs, and Seurat DESeq2. Bar heights represent the number of shared DE genes (intersection size), while horizontal bars indicate the total number of DE genes per method (set size).

Figure S5.

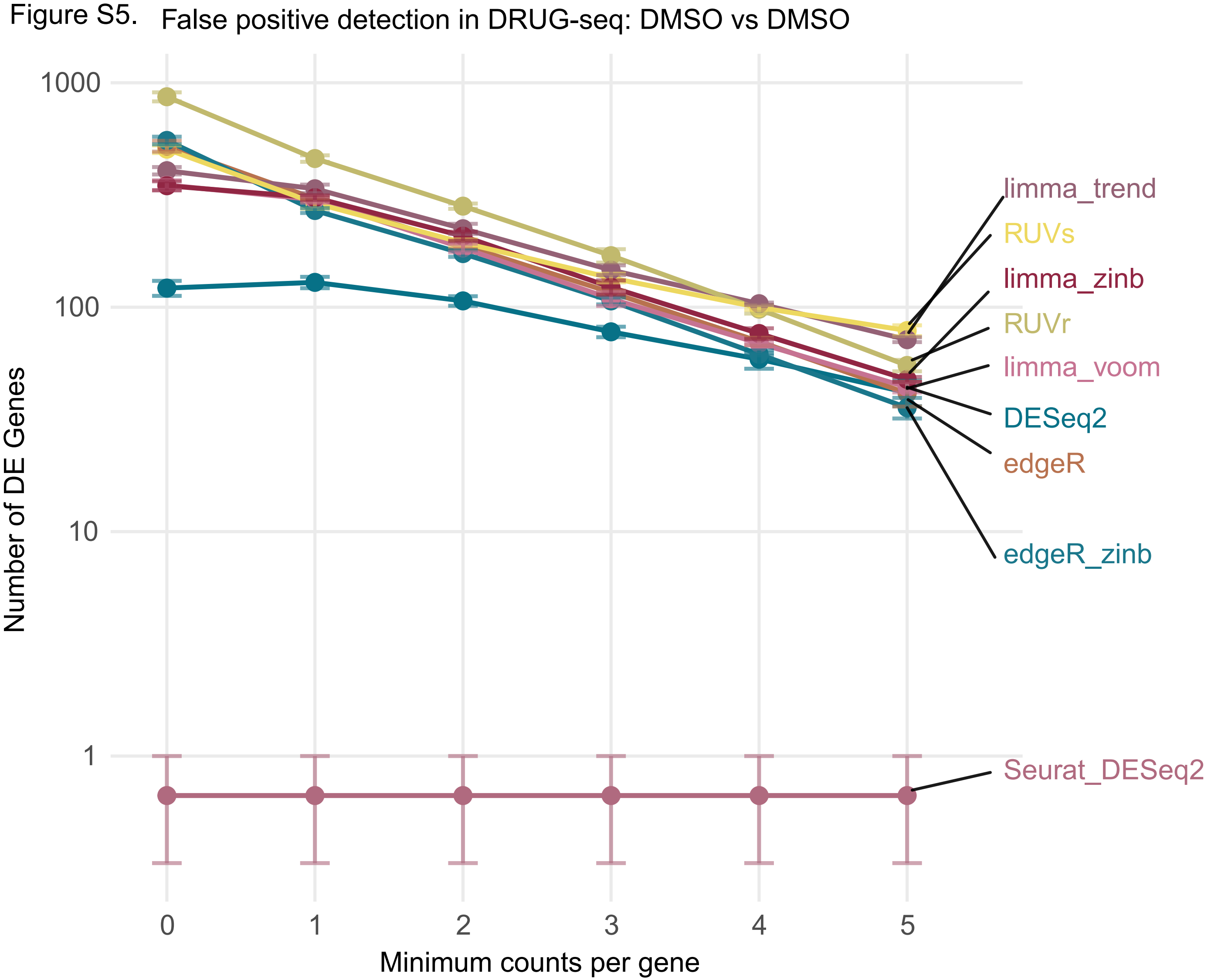

Supplementary Figure S5. False positive detection rates across normalization and modeling methods. Line plot shows the number of genes identified between two DMSO control groups in DRUG-seq data with nominal p-value < 0.05, and |log2FC| > 1, across increasing minimum read count thresholds per gene.

Figure S6.

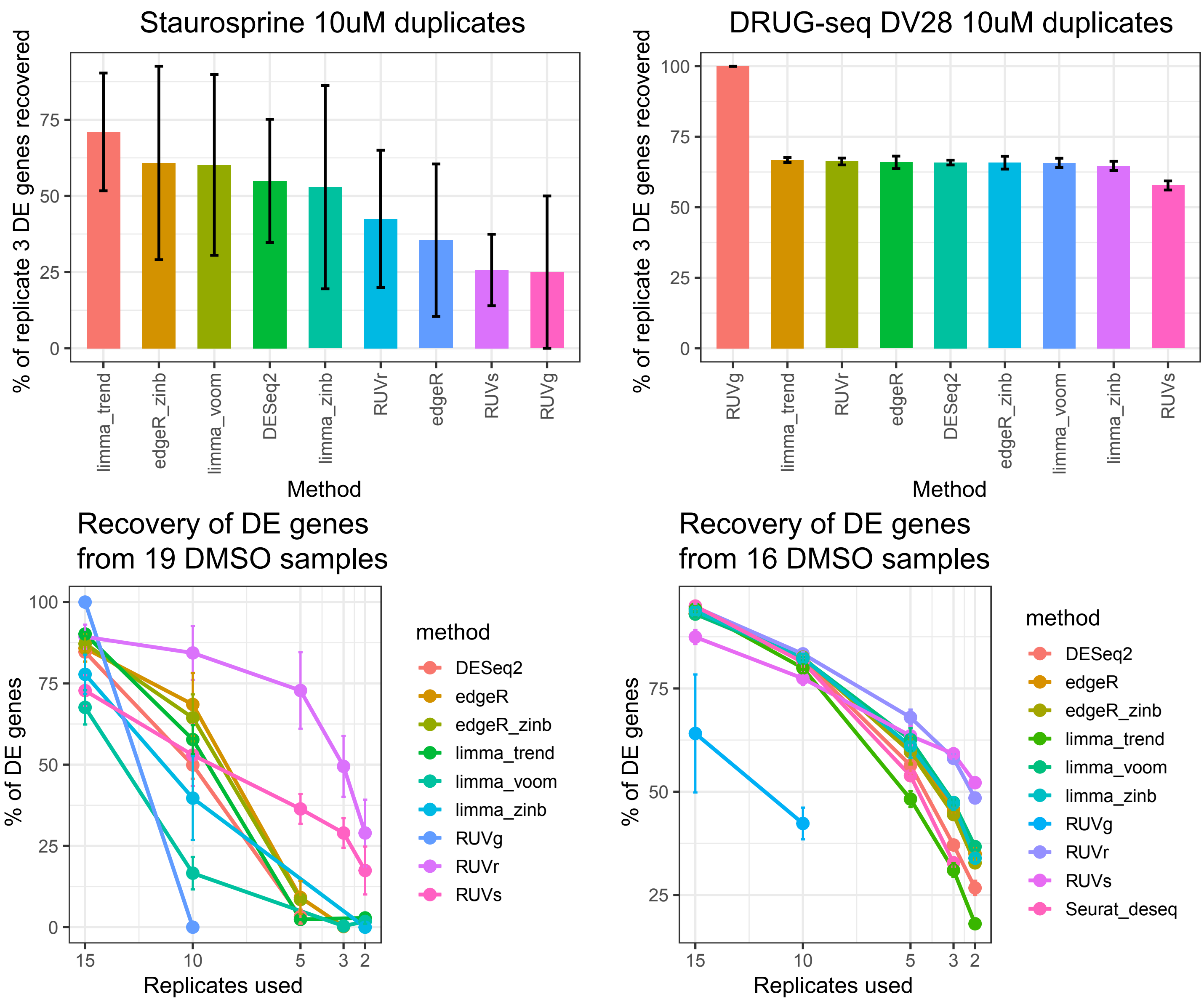

Supplementary Figure S6. Reproducibility of differential expression detection upon reduction of number of replicates. Bar graph in top panels represent percent of DE genes from duplicates (Camptothecin vs DMSO, 3x n=2) overlapping DE genes from a triplicate (Camptothecin vs DMSO, 1x n=3) across statistical models, |log2FC|>1 and FDR<0.05. Error bars represent standard error (n=3). Line graphs in bottom panels show relationship between the number of control replicates and DE gene yield across statistical models. For each set of replicates, a comparison was made against the whole 19 (MAC-seq) or 16 (DRUG-seq) DMSO replicates.
